# Spatial dynamics and the basic reproduction number of the 1991–1997 Cholera epidemic in Peru

**DOI:** 10.1371/journal.pntd.0008045

**Published:** 2020-07-14

**Authors:** Alexandra Smirnova, Natalie Sterrett, Oscar J. Mujica, César Munayco, Luis Suárez, Cécile Viboud, Gerardo Chowell

**Affiliations:** 1 Department of Mathematics and Statistics, Georgia State University, Atlanta, Georgia, United States of America; 2 Department of Population Health Sciences, School of Public Health, Georgia State University, Atlanta, Georgia, United States of America; 3 Pan American Health Organization (PAHO), Washington DC, United States of America; 4 Centro Nacional de Epidemiología, Prevención y Control de Enfermedades, Lima, Peru; 5 Division of International Epidemiology and Population Studies, Fogarty International Center, National Institutes of Health, Bethesda, Maryland, United States of America; Institute for Disease Modeling, UNITED STATES

## Abstract

After being cholera free for over 100 years, Peru experienced an unprecedented epidemic of *Vibrio cholerae* O1 that began in 1991 and generated multiple waves of disease over several years. We developed a mechanistic transmission model that accounts for seasonal variation in temperature to estimate spatial variability in the basic reproduction number ($R_{0}$), the initial concentration of vibrios in the environment, and cholera reporting rates. From 1991-1997, cholera spread following a multi-wave pattern, with weekly incidence concentrated during warm seasons. The epidemic first hit the coastal departments of Peru and subsequently spread through the highlands and jungle regions. The correlation between model predictions and observations was high (range in *R*^2^: 58% to 97%). Department-level population size and elevation explained significant variation in spatial-temporal transmission patterns. The overall *R*_0_ across departments was estimated at 2.1 (95% CI: 0.8,7.3), high enough for sustained transmission. Geographic-region level $R_{0}\text{s}$ varied substantially from 2.4 (95% CI: 1.1, 7.3) for the coastal region, 1.9 (0.7, 6.4) for the jungle region, and 1.5 (0.9, 2.2) for the highlands region. At the department level, mean $R_{0}$ ranged from 0.8 to 6.9. Department-level $R_{0}\text{s}$ were correlated with overall observed attack rates (Spearman *ρ* = 0.59, *P* = 0.002), elevation (*ρ* = −0.4, *P* = 0.04), and longitude (*ρ* = −0.6, *P* = 0.004). We find that both $R_{0}$ and the initial concentration of vibrios were higher in coastal departments than other departments. Reporting rates were low, consistent with a substantial fraction of asymptomatic or mild cases associated with the El Tor cholera biotype. Our results suggest that cholera vibrios, autochthonous to plankton in the natural aquatic environment, may have triggered outbreaks in multiple coastal locations along the Pacific coast of Peru. Our methodology could be useful to investigate multi-wave epidemics of cholera and could be extended to conduct near real-time forecasts and investigate the impact of vaccination strategies.

## Author summary

After being cholera free for over 100 years and in the absence of an effective vaccine against the disease, Peru experienced one of the worst multi-wave epidemics in South American history from 1991-1997. Here we applied statistical and mathematical modeling to a weekly spatio-temporal dataset of cholera cases to investigate transmission patterns and variation in the basic reproduction number. Our findings indicate that the epidemic first hit the coastal departments of Peru and subsequently spread through the highlands and jungle regions. There were 3−4 consecutive well-defined waves concentrated during the warm seasons. Cholera transmission potential and the initial concentration of vibrios were higher in coastal departments compared to other departments. Moreover, reporting rate estimates were low, consistent with the significant fraction of asymptomatic or mild cases that is associated with the El Tor cholera biotype. Our results suggest that cholera vibrios, autochthonous to plankton in the natural aquatic environment, may have been present in multiple coastal locations, possibly leading to multiple disease introductions along the Pacific coast of Peru.

## Introduction

Largely transmitted through contaminated food or water, *Vibrio cholerae* continues to generate outbreaks of acute gastrointestinal illness particularly in lower-income countries with poor sanitary infrastructure; it currently affects 1.3 to 4 million people annually worldwide [1]. Ecological studies suggest that warm brackish waters are an ideal reservoir for *V. cholerae*, where it can attach to aquatic organisms such as shellfish and zooplankton [2, 3]. The bacterium thrives in warm water bodies, which increases the risk of cholera epidemics in susceptible populations [4]. Although our understanding of the epidemiological and clinical characteristics of this pathogen has advanced considerably [3], quantitative analysis of major historical epidemics in immunologically naive populations is needed to elucidate the drivers of transmission.

Historically, cholera epidemics have been associated with one of two biotypes, both of which belong to *V. cholerae* serogroup O1 [4]. Up to 1960, epidemic cholera was primarily caused by the classical O1 biotype, but it was subsequently replaced by *V. cholerae* El Tor, marking the beginning of the seventh cholera pandemic in 1961 [4]. Infection with the El Tor biotype is associated with frequent mild or asymptomatic infection and the ability to survive in both human hosts and the environment—a known evolutionary tradeoff [5]. Several case-control studies have shown asymptomatic controls to have 29% to 34% seroconversion, implying the disease spreads effectively yet rarely causes significant symptoms [6, 7]. However, rapid onset of watery diarrhea, vomiting, cramping, and subsequent dehydration occurs in approximately one out of every 10-50 infected individuals [4]. If left untreated, it can lead to shock, renal failure, and eventually death [4]. Intravenous fluids and oral rehydration, along with appropriate use of antibiotics, can reduce case-fatality to less than one percent [4].

*V. cholerae* El Tor was isolated for the first time from deceased pilgrims returning from La Meca in El Tor control station, Egypt, in 1905. The ongoing seventh cholera pandemic originated in the Bay of Bengal, involving at least three separate waves of infection with the El Tor strain [8]. The pandemic originated in Indonesia in 1961 [9], from there spreading through India (1964) [10], Africa (1970) [11], Southern Europe (1970) [12], and South America (1991) [13], returning to the Americas after more than 100 years [14]. In the absence of an effective vaccine against cholera in the early 1990s [15], Peru experienced one of the worst multi-year epidemics in South American history [16, 17, 18]. Between late January 1991 and December 1993, the epidemic caused about a million cases and almost 9000 deaths [19]. Although it was initially speculated that a Chinese ship imported the disease through an infected crew member [20], it is more likely that the culprit pathogen was already widespread in the local environment, implicating an environmental/water-borne source [21].

A limited picture of the impact of the epidemic in Peru can be gleaned through largely descriptive local reports of the epidemic and several case-control studies [16, 15, 22, 23, 6, 7]. For instance, the first cases of cholera in Peru were reported in the central coast in late January 1991 [24], with subsequent cases reported almost simultaneously in coastal cities farther north [25]. Cholera then spread rapidly to the rest of the country [13], was widespread in sea and river waters as well as in municipal sewage and seafood [26], and continued to generate outbreaks for several more years [16]. It has been estimated that in 1991, between 3 and 11 million individuals were infected, 2.4 million individuals developed cholera diarrhea, 322, 562 individuals sought care, and 2, 909 Peruvians died [27]. A quantitative analysis of the spatial-temporal spread of the devastating cholera epidemic in Peru is useful to elucidate the drivers of transmission in an immunologically naive population at a time when an effective vaccine was unavailable.

Mathematical modeling have helped quantify the transmission rates and reproduction numbers of cholera epidemics in various locations, which can inform control measures [28, 29, 30, 31, 32]. However, there is a scarcity of estimates of the basic reproduction number ($R_{0}$) in essentially naive populations, and the 1991-1997 cholera epidemic in Peru represents an interesting case study [16, 17]. In this paper, we combine dynamic modeling based on ordinary differential equations and statistical estimation methods along with a dataset of weekly cholera cases across departments of Peru to generate estimates of the spatial-temporal variation in the basic reproduction number at three spatial scales (i.e., national, regional, and departmental) and assess the pattern of spread vis-à-vis environmental and socio-demographic variables.

### Setting

Peru is located on the Pacific coast, sharing borders with Bolivia, Brazil, Chile, Colombia, and Ecuador. In 1990, Peru’s population exceeded 22 million, and was heterogeneously distributed across a surface area of 1 285 220 km^2^ comprising 25 administrative units (24 departments and 1 constitutional province hereafter referred to as 25 departments; S1 Fig). Peru is characterized by three geographic zones with diverse climates: the dry, desert western coast along the Pacific Ocean, the temperate Andean highlands or more central departments, and the tropical Amazon jungle toward the East. In a country like Peru, we expect variability in cholera dynamics across different geographic regions, as temperature is known to influence transmission [2].

At the time of the epidemic, Peru struggled with access to healthcare, environmental sanitation, and political and economic issues [15], which complicated the implementation of control interventions necessary to mitigate a large epidemic [20]. Inadequate water treatment and deficiencies in water storage systems have also been documented throughout the country [15, 33]. Further, between 1987 and 1990, Peru’s economy had declined dramatically, with the gross domestic product dropping by 23%, the per capita income and purchase power falling by one third, and public expenditures being reduced by 52% in health and 28% in education [34]. Given the great magnitude of the epidemic, the healthcare infrastructure was overrun by the large number of cases presenting to clinics and hospitals [35].

## Materials and methods

### Epidemiologic data

Peru’s General Office of Epidemiology launched the epidemiological surveillance system along with the Peruvian Field Epidemiology Training Program in 1989, not long before the cholera epidemic hit Peru [36, 37, 38, 39]. This surveillance system generates weekly surveillance reports across 25 departments by relying on a network of over 6,000 geographically distributed health-care units reporting cases [40]. During the 1991-1997 cholera epidemic, epidemiological surveillance included both confirmed and suspected cases. A suspected case was defined as any patient older than five years presenting with acute and watery diarrhea [41, 42], a case definition that remained stable during the epidemic. Confirmed cases were laboratory-confirmed with *Vibrio cholerae* 01 El Tor Inaba [43, 41]. Time series of weekly incidences across departments are available in a public repository [44].

### Environmental and geographic data

Weekly temperature time series were obtained from the European Centre for Medium-Range Weather Forecasts’s ERA-Interim atmospheric reanalysis archive from 1991 to 1997. The ERA-interim model allows estimation of daily minimum, mean, and maximum temperatures by department [45], which we used to assess the relationship between case incidence and temperature at the department level. We also collected several geographic variables including elevation, illiteracy rate, population size, latitude, and longitude, which were used in spatial-temporal analysis [46] (S2 Fig). These datasets are available online [44].

### The basic reproduction number ($R_{0}$)

The basic reproduction number ($R_{0}$) is a key epidemiological metric for assessing the transmission potential of infectious disease outbreaks [47]. $R_{0}$ is typically defined as the average number of secondary cases generated by a primary case in an entirely susceptible population [47]. In general, if $R_{0}>1$ an epidemic is expected, while disease transmission cannot be sustained if $R_{0}<1$. In the context of infectious disease transmission that is partly driven by an environmental component (e.g., temperature), the actual value of $R_{0}$ depends on time. Thus, we denote $R_{0}\left(t\right)$ as the time dependent basic reproduction number in the absence of susceptible depletion. In our study, we define the mean $R_{0}$ as the average of $R_{0}\left(t\right)$ during our study period. We compare mean $R_{0}$ estimates at three different spatial scales: national, regional (e.g., coastal, jungle, highlands), and department.

### Mathematical model of cholera transmission dynamics

We estimated the mean $R_{0}$ at the department level using a mechanistic model of cholera transmission together with a novel parameter estimation approach. We adapted a compartmental dynamic model that has been previously used to estimate transmission potential of cholera epidemics [28, 29, 30, 48, 49]. Our cholera model, consisting of 4 equations (Eqs (0.1)–(0.4)) and 8 parameters, incorporates the effects of local temperature fluctuations on the environmental transmission rate (Table 1). In addition to susceptible (*S*), infectious (*I*), and removed (*R*) compartments, this model includes a compartment (*B*) that models the concentration of vibrios in the environment (e.g., water supply). Hence, the model accounts for two transmission pathways: 1) cholera exposure from the contaminated environment/water supply and 2) human-to-human transmission via close contact with infectious individuals.

**Table 1:** Parameter definitions and baseline values associated with the mechanistic cholera transmission model.

Symbol	Definition	Value	Reference
*μ*	Natural birth & death rate	1/(60 ⋅ 52) weeks^−1^	
*κ*	50% infectious dose	10^6^ ⋅mL^−1^	[48]
*γ*	Recovery rate	7/5 weeks^−1^	[51]
λ	Rate of contribution of vibrios from infected individuals to environment	70 ⋅mL^−1^⋅weeks^−1^	[48]
*δ*	Death rate of vibrios in environment	7/30 weeks^−1^	[14]
*β*_*h*_	Human to human transmission rate	Estimated	
*β*_*e*1_	Baseline environmental transmission rate	Estimated	
*β*_*e*2_	Relative environmental transmission forcing	Estimated	
*B*_1_	Initial concentration of vibrios	Estimated	
*ψ*	Reporting rate	Estimated	

In this model, individuals in a population of size *N* are born and die at rate *μ*. Susceptible individuals can be infected through the environment with time-dependent transmission rate *β*_*e*_(*t*) or through human contact with transmission rate *β*_*h*_. Therefore, they move from susceptible to infectious classes at rates *β*_*e*_(*t*)*B*/(*B* + *κ*) (where *κ* is the 50% infectious dose in the environment and *B* is the current concentration of vibrios in the environment) [28] and *β*_*h*_*I*. Vibrios are shed by infectious individuals into the environment at rate λ, and then die at rate *δ*. Infected individuals are assumed to recover and acquire protective immunity for the duration of the entire epidemic period at rate *γ* [50]. The overall transmission dynamics can be mathematically described by the following set of nonlinear differential equations:

$$\begin{array}{ccc} \frac{dS}{dt} & = & \mu N-\beta_{h}S\left(t\right)I\left(t\right)-\beta_{e}\left(t\right)S\left(t\right)\frac{B(t)}{B(t)+\kappa }-\mu S\left(t\right) \end{array}$$ (0.1)

$$\begin{array}{ccc} \frac{dI}{dt} & = & \beta_{h}S\left(t\right)I\left(t\right)+\beta_{e}\left(t\right)S\left(t\right)\frac{B(t)}{B(t)+\kappa }-\mu I\left(t\right)-\gamma I\left(t\right) \end{array}$$ (0.2)

$$\begin{array}{ccc} \frac{dR}{dt} & = & \gamma I(t)-\mu R(t) \end{array}$$ (0.3)

$$\begin{array}{ccc} \frac{dB}{dt} & = & \lambda I(t)-\delta B(t), \end{array}$$ (0.4)

with initial conditions

$$\begin{array}{c} S\left(0\right)=N-C_{1},\;I\left(0\right)=C_{1},\;R\left(0\right)=0,\;B\left(0\right)=B_{1}, \end{array}$$ (0.5)

where *N* is the population size for a given department in Peru, *C*_1_ is the number of cases observed in the first week in each department divided by a reporting rate, and *B*_1_ is the initial concentration of vibrios in the environment. We assume that reported data, *D*, is available for the weekly incidence cases subject to an unknown reporting rate, *ψ*. In our model, the weekly temperature variation, *T*(*t*), directly influences the cholera transmission rate from the environment. To reflect that, *β*_*e*_(*t*) is further broken down into two components: *β*_*e*_(*t*) = *β*_*e*1_ + *β*_*e*2_*T*(*t*), where *T*(*t*) represents the mean temperature at time *t* for the corresponding department. According to (0.1)–(0.5), the cumulative number of human cases, *C*(*t*), satisfies the following differential equation:

$$\begin{array}{ccc} \frac{dC}{dt} & = & \beta_{h}S\left(t\right)I\left(t\right)+\beta_{e}\left(t\right)S\left(t\right)\frac{B(t)}{B(t)+\kappa }. \end{array}$$ (0.6)

By fitting $\psi \frac{dC}{dt}$ to the reported incidence data, *D*(*t*), we estimate five system parameters: *β*_*h*_, *β*_*e*1_, *β*_*e*2_ (the three transmission coefficients), *B*_1_ (the initial concentration of vibrios in the environment), and *ψ* (the reporting rate). The reporting rate, *ψ*, is a scaling factor used to adjust for possible underreporting of cases, owing to, for instance, a large proportion of asymptomatic cholera cases [14]. Table 1 includes all model parameters and their definitions, as well as the values chosen for the parameters that are pre-estimated.

For this compartmental model, the time-dependent basic reproduction number ($R_{0}\left(t\right)$) is given by [28]:

$$\begin{array}{c} R_{0}\left(t\right)=\frac{N}{\delta \kappa (\gamma +\mu )}\left(\lambda \beta_{e}\left(t\right)+\delta \kappa \beta_{h}\right) \end{array}$$ (0.7)

The effective reproduction number at calendar time *t* accounts for the depletion of susceptible individuals and is given by $R_{e}\left(t\right)$:

$$\begin{array}{c} R_{e}\left(t\right)=\frac{N}{\delta \kappa (\gamma +\mu )}\left(\lambda \beta_{e}\left(t\right)+\delta \kappa \beta_{h}\right)s^{*}\left(t\right), \end{array}$$ (0.8)

where s*(*t*) is the fraction of susceptible individuals in the population at time *t*. In the next section we describe the algorithm for stable estimation of the unknown disease parameters, *β*_*h*_, *β*_*e*1_, *β*_*e*2_, *B*_1_, and *ψ*, for each department. Unlike most previously used inversion schemes, this approach does not rely on numerical solution of a nonlinear system (0.1)–(0.5) at every step of the iterative process [52]. Instead, it effectively combines analytical and numerical optimization tools in order to reduce computational complexity and the resulting noise propagation in the recovered parameter values.

### Parameter identifiability

Prior studies have underscored parameter identifiability issues related to infectious disease transmission models based on ordinary-differential equations [53, 54, 55]. Lack of identifiability, or non-identifiability, which is evident when parameter estimates are associated with large uncertainties, may be attributed to the model structure (structural identifiability) or due to the lack of information in a given dataset, which could be associated with the number of observations and the spatial granularity of the data [53]. Because the time series of reported incident cases stems from the aggregation of sub-epidemics associated with multiple exposure types [56], it can give rise to indistinguishable epidemic waves. In the context of cholera transmission dynamics, it is difficult to disentangle the contributions of different transmission routes (e.g., environmental exposure versus cases stemming from person-to-person transmission) [54]. While it is difficult to estimate the transmission coefficients (*β*_*h*_, *β*_*e*1_, *β*_*e*2_), we show that it is still feasible to derive composite $R_{0}$ estimates, which is consistent with prior studies [53, 54]. We report estimates for three key parameters and their associated uncertainty at the level of departments: 1) $R_{0}$, 2) the initial concentration of vibrios in the environment, and 3) the reporting rate.

### Mathematical preliminaries and optimization algorithm

In this subsection we present our novel problem-oriented parameter estimation method, which takes full advantage of the available incidence data in the construction of a parameter-to-data map for the least squares problem (LSP). Even though the LSP still needs to be solved by a regularized trust-region nonlinear optimization algorithm, this algorithm is no longer combined with a numerical solution of the nonlinear system of differential equations at every step of the iterative process, which differs from standard estimation methods. As a result, the total computational error and the propagation of noise in the estimated parameters are significantly reduced, hence bringing accuracy and stability in the inversion scheme. Moreover, our effective use of incidence data while analytically solving the ODE system (0.1)–(0.5) and subsequently discretizing its solution makes it possible to form a parameter-to-data map, which, despite being nonlinear in *ψ* and *B*_1_, turns out to be linear in all three transmission parameters, *β*_*h*_, *β*_*e*1_, and *β*_*e*2_, thus further reducing computational complexity of the proposed method.

Indeed, replacing the force of infection, $\beta_{h}S\left(t\right)I\left(t\right)+\beta_{e}\left(t\right)S\left(t\right)\frac{B(t)}{B(t)+\kappa }$, with $\frac{dC}{dt}$ in the first two equations of compartmental model (0.1)–(0.5), one gets linear nonhomogeneous ordinary differential equations (ODEs) for *S*(*t*) and *I*(*t*), respectively:

$$\begin{array}{ccc} \frac{dS}{dt} & = & -\mu S\left(t\right)-\frac{dC}{dt}+\mu N \end{array}$$ (0.9)

$$\begin{array}{ccc} \frac{dI}{dt} & = & -\left(\mu +\gamma \right)I\left(t\right)+\frac{dC}{dt}. \end{array}$$ (0.10)

Taking into account initial conditions (0.5), one arrives at the following analytical solutions to (0.9) and (0.10), respectively:

$$\begin{array}{ccc} S(t) & = & N-\text{exp}\left(-\mu t\right)C_{1}-\int_{0}^{t}\text{exp}\left(-\mu \left(t-s\right)\right)C^{\prime }\left(s\right)\;ds \end{array}$$ (0.11)

$$\begin{array}{ccc} I(t) & = & \text{exp}\left(-\left(\mu +\gamma \right)t\right)C_{1}+\int_{0}^{t}\text{exp}\left(-\left(\mu +\gamma \right)\left(t-s\right)\right)C^{\prime }\left(s\right)\;ds. \end{array}$$ (0.12)

To derive the equation for *B*(*t*) in terms of *C*′(*t*), one can first write *B*(*t*) as

$$\begin{array}{ccc} B(t) & = & \text{exp}\left(-\delta t\right)B_{1}+\lambda \int_{0}^{t}\text{exp}\left(-\delta \left(t-s\right)\right)I\left(s\right)\;ds. \end{array}$$ (0.13)

Substituting (0.12) for *I*(*s*) into (0.13) and integrating by parts to eliminate the inner integral, one arrives at the following identity:

$$\begin{array}{ccc} B(t) & = & \text{exp}\left(-\delta t\right)B_{1}+\frac{\lambda C_{1}}{\mu +\gamma -\delta }\left[\text{exp}\left(-\delta t\right)-\text{exp}\left(-\left(\mu +\gamma \right)t\right)\right]\\ & + & \frac{\lambda }{\mu +\gamma -\delta }\int_{0}^{t}C^{\prime }\left(s\right)\left[\text{exp}\left(-\delta \left(t-s\right)\right)-\text{exp}\left(-\left(\mu +\gamma \right)\left(t-s\right)\right)\right]\;ds. \end{array}$$ (0.14)

The next step in our algorithm is to obtain discrete analogs of *S*(*t*), *I*(*t*), and *B*(*t*) at the mesh points *t*_1_, *t*_2_,…, *t*_*m*_, where *t*_*i*_ = *i* − 1, *i* = 1, 2, …, *m*, and *t*_1_ = 0 is the first week of the outbreak. Recall that $\psi \frac{dC}{dt}$ must be fitted to the reported incidence data, *D*(*t*). Considering that, we replace *C*_1_ with *D*_1_/*ψ* and *C*′(*s*) with *D*(*s*)/*ψ* under each integral in (0.11), (0.12), and (0.14). Given discrete data, *D* = [*D*_1_, *D*_2_, …, *D*_*m*_]^⊤^, reported weekly, we interpolate *D* as follows:

$$\begin{array}{c} D\left(0\right)=D\left(t_{1}\right)=D_{1},\;\text{and}\;D\left(t\right)=D_{j+1}\;\text{for}\;t\in \left(t_{j},t_{j+1}\right],\;j=1,2,...,m-1. \end{array}$$ (0.15)

From (0.15), one concludes that *S*_1_ = *N* − *D*_1_/*ψ* and for *i* = 2, 3, …, *m*,

$$\begin{array}{ccc} S_{i} & = & N-\frac{\text{exp}\left(-\mu t_{i}\right)D_{1}}{\psi }-\sum_{j=1}^{i-1}\frac{D_{j+1}}{\psi }\int_{t_{j}}^{t_{j+1}}\text{exp}\left(-\mu \left(t_{i}-s\right)\right)\;ds. \end{array}$$ (0.16)

Evaluating integrals analytically and substituting *i* − 1 for *t*_*i*_, one gets

$$\begin{array}{ccc} S_{1}\left[\psi \right] & = & N-D_{1}/\psi \\ S_{i}\left[\psi \right] & = & N-\frac{\text{exp}\left(-\mu \left(i-1\right)\right)D_{1}}{\psi }-\frac{1}{\mu \psi }\sum_{j=1}^{i-1}D_{j+1}\left[\text{exp}\left(-\mu \left(i-j-1\right)\right)-\text{exp}\left(-\mu \left(i-j\right)\right)\right], \end{array}$$ (0.17)

*i* = 2, 3, …, *m*. Likewise, identities (0.12) and (0.14) yield

$$\begin{array}{ccc} I_{1}\left[\psi \right] & = & D_{1}/\psi \\ I_{i}\left[\psi \right] & = & \frac{\text{exp}\left(-\left(\mu +\gamma \right)\left(i-1\right)\right)D_{1}}{\psi }\\ & + & \frac{1}{(\mu +\gamma )\psi }\sum_{j=1}^{i-1}D_{j+1}\left[\text{exp}\left(-\left(\mu +\gamma \right)\left(i-j-1\right)\right)-\text{exp}\left(-\left(\mu +\gamma \right)\left(i-j\right)\right)\right], \end{array}$$ (0.18)

and

$$\begin{array}{ccc} \;B_{i}\left[\psi ,B_{1}\right] & = & \text{exp}\left(-\delta \left(i-1\right)\right)B_{1}+\frac{\lambda D_{1}}{(\mu +\gamma -\delta )\psi }\left[\text{exp}\left(-\delta \left(i-1\right)\right)-\text{exp}\left(-\left(\mu +\gamma \right)\left(i-1\right)\right)\right]\\ & + & \frac{\lambda }{(\mu +\gamma -\delta )\psi }\sum_{j=1}^{i-1}D_{j+1}[\frac{\text{exp}(-\delta (i-j-1))-\text{exp}(-\delta (i-j))}{\delta }\\ & - & \frac{\text{exp}(-(\mu +\gamma )(i-j-1))-\text{exp}(-(\mu +\gamma )(i-j))}{\mu +\gamma }]. \end{array}$$ (0.19)

*i* = 2, 3, …, *m*. This implies that estimation of the unknown parameters, *β*_*h*_, *β*_*e*1_, *β*_*e*2_, *B*_1_, *ψ*, can now be cast as the following nonlinear least squares problem:

$$\begin{array}{c} \min_{\beta_{h},\beta_{e1},\beta_{e2},B_{1},\psi }\;\;\frac{1}{2}\;{\left\|\psi S\left[\psi \right]\{\beta_{h}I\left[\psi \right]+\left(\beta_{e1}+\beta_{e2}T\right)\frac{B[\psi ,B_{1}]}{B[\psi ,B_{1}]+\kappa }\}-D\right\|}^{2} \end{array}$$ (0.20)

$$\begin{array}{c} =\min_{\beta_{h},\beta_{e1},\beta_{e2},B_{1},\psi }\;\;\frac{1}{2}\;\sum_{i=1}^{m}{\left(\psi S_{i}\left[\psi \right]\{\beta_{h}I_{i}\left[\psi \right]+\left(\beta_{e1}+\beta_{e2}T_{i}\right)\frac{B_{i}\left[\psi ,B_{1}\right]}{B_{i}\left[\psi ,B_{1}\right]+\kappa }\}-D_{i}\right)}^{2}, \end{array}$$ (0.21)

where *D* = [*D*_1_, *D*_2_, …, *D*_*m*_]^⊤^ and *T* = [*T*_1_, *T*_2_, …, *T*_*m*_]^⊤^ are reported data sets for incident cases and mean weekly temperature fluctuations, respectively, and the expressions for *S*_*i*_[*ψ*], *I*_*i*_[*ψ*], and *B*_*i*_[*ψ*, *B*_1_] are given by (0.17), (0.18) and (0.19).

After the five unknown parameters have been recovered from the corresponding epidemic data sets using our proposed optimization algorithm, 500 additional incidence curves are generated via parametric bootstrap [57] in order to quantify uncertainty in the estimated parameters and derive 95% confidence intervals (S1 Table). Matlab code is available upon request from the authors.

### Spatial analysis

We used Spearman’s rho (*ρ*) and multiple linear regression to assess the relationship between department-level predictors, cumulative incidence, week of epidemic onset, estimates of basic reproduction numbers, reporting rates, and initial concentrations of vibrios. For each department, epidemic onset was defined as the first week with reported cholera cases. Additionally, we generated maps of cholera attack rates by year. Maps were created using the choropleth package in R.

### Spatial autocorrelation

Spatial autocorrelation is a measure of similarity of nearby observations. We assessed spatial autocorrelation of attack rates (cumulative cases during the study period) across departments using Moran’s I statistic [58]. Moran’s I is calculated using a nearest neighbor matrix *w*_*ij*_ of the 25 spatial units where *w*_*ij*_ = 1 when departments *i* and *j* share a border. All other entries are equal to zero. The statistic is calculated as in Eq (0.22), where *N* is the number of departments, *x*_*i*_ is the cholera incidence in department *i*, $\bar{x}$ is the mean cholera incidence across departments, and *W* is the sum of entries in matrix *w*_*ij*_:

$$\begin{array}{c} I=\frac{N\sum_{i=1}^{N}\sum_{j=1}^{N}w_{ij}\left(x_{i}-\bar{x}\right)\left(x_{j}-\bar{x}\right)}{W\sum_{i=1}^{N}{\left(x_{i}-\bar{x}\right)}^{2}}. \end{array}$$ (0.22)

To determine significance of Moran’s I, we used a nonparametric random data permutation test [59]. We sampled 10,000 random permutations of Peruvian departments given our observed data, generating a reference distribution of Moran’s statistics under the null hypothesis of no spatial autocorrelation. P-values were calculated as the probability of obtaining the observed Moran’s I or a more extreme value from the reference distribution [60].

### Spatial heterogeneity (Gini index)

We also quantified heterogeneity in attack rates using the Lorenz curve and Gini index [61, 62]. The Lorenz curve is a graphical display of the cumulative proportion of cholera cases against the cumulative proportion of population. Under the assumption of homogeneity, the distributions will be balanced, and the Lorenz curve will fall on the diagonal. As heterogeneity in attack rates increases, the curve will become farther from this reference line. The Gini index is a summary measure of heterogeneity, calculated as the ratio of the area between the Lorenz curve and the reference line to the total area beneath the reference line. The Gini index ranges from 0 to 1, with a larger value indicating greater spatial heterogeneity.

## Results

**Fig 1:**
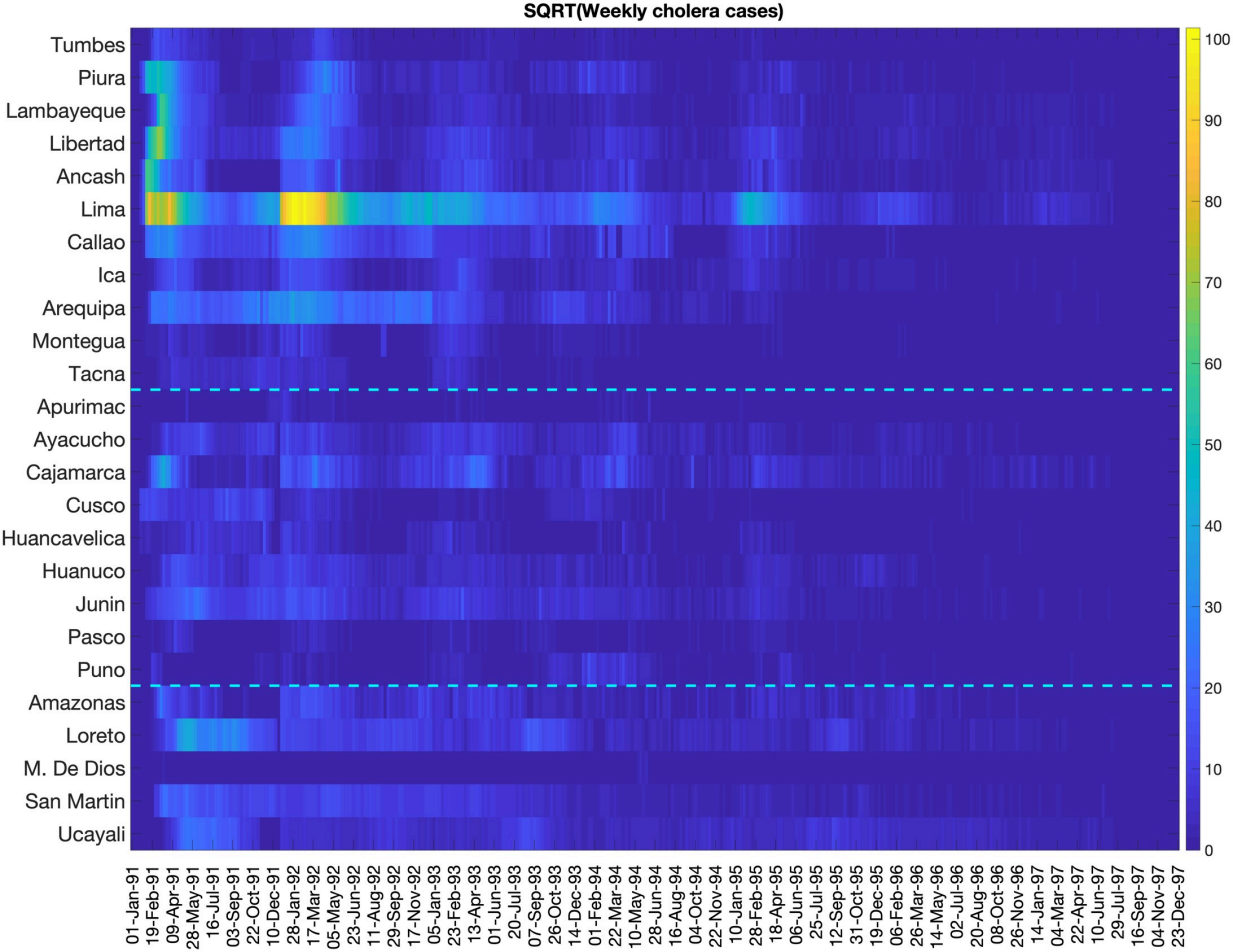
Color scale image of weekly cholera cases by department. Weekly cases have been square root transformed to reduce variability in the amplitude of the time series while dashed lines separate the coast, highland, and jungle regions. The epidemic hit the coastal departments early, with the highest case counts concentrated in this region.

**Fig 2:**
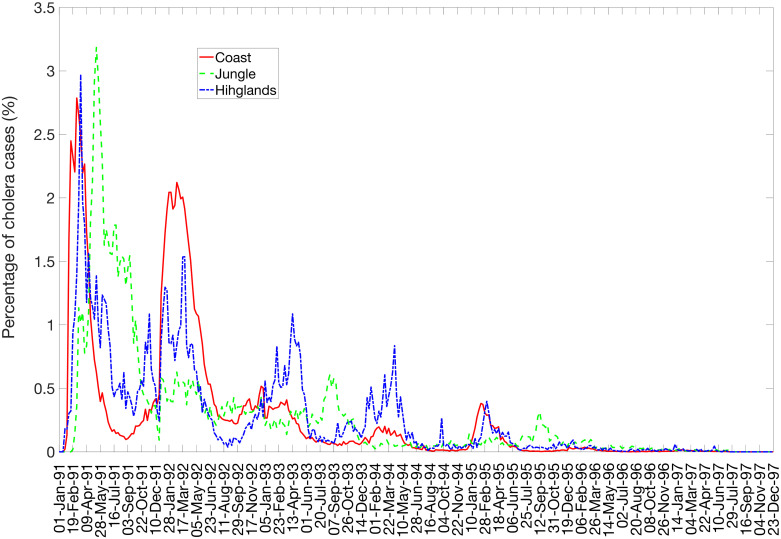
Weekly incidence of cholera cases in Peru by region, January 1991 through December 1997. Curves represent the national and regional weekly proportions of total cases reported during 1991-1997.

**Fig 3:**
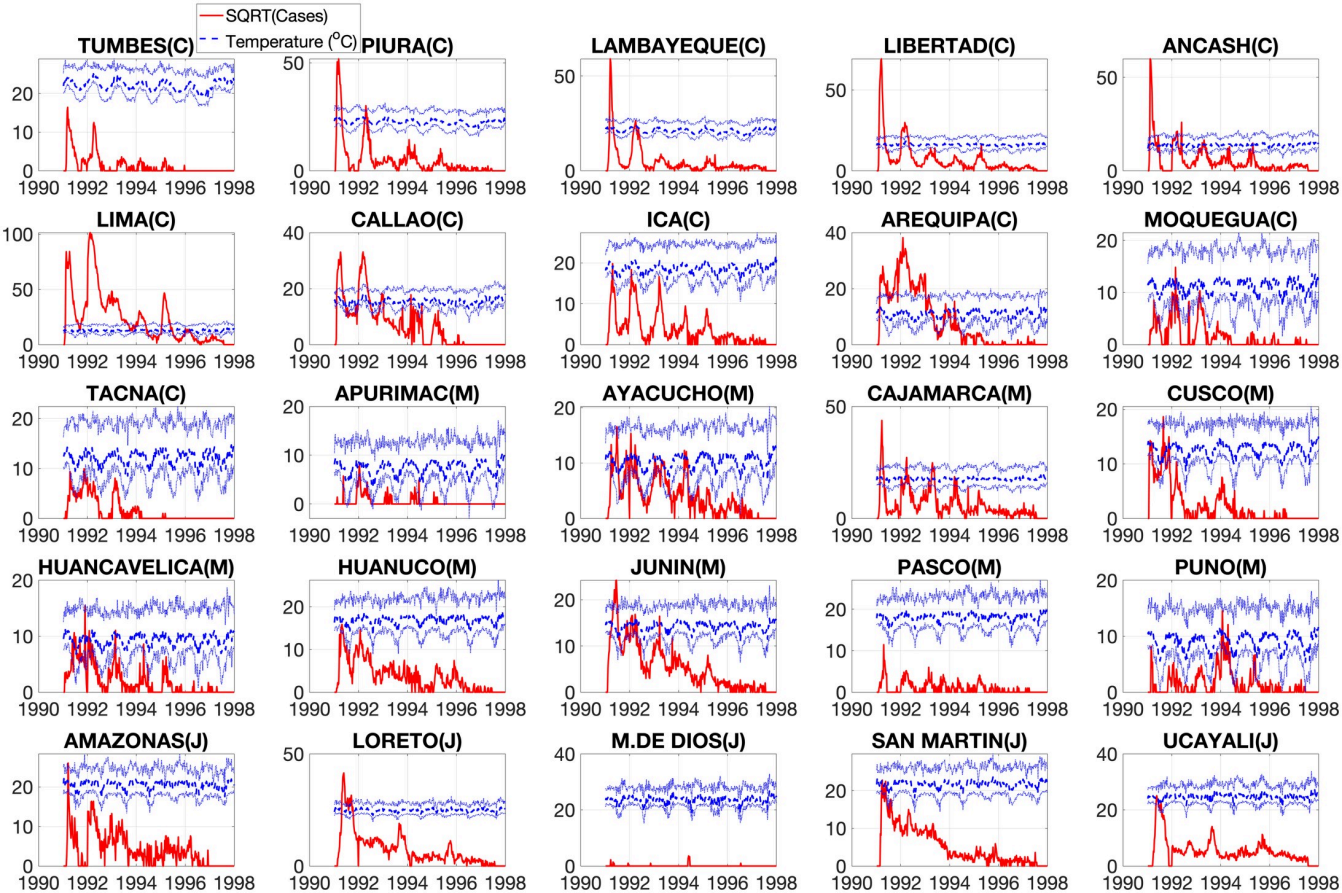
Time series of weekly cholera cases (red solid line) and temperature (blue curves; minimum, mean, and maximum temperature) across 25 departments during the 1991-1997 cholera epidemic in Peru.

The first cholera cases were reported to the Peruvian Ministry of Health in late January of 1991. Infection spread so rapidly that by late February, the coastal departments had already received the brunt of the epidemic, though infection would resurge in subsequent years (Fig 1). As seen in Fig 2, the epidemic progressed in three spatial waves, first hitting the coast and subsequently spreading through the highlands and jungle regions. Most of the cases occurred during the first three years of the epidemic (S3 Fig). Moreover, our results indicate that larger populations tended to have earlier epidemic onset (Spearman *ρ* = −0.519, *P* < 0.01), with the epidemic thereafter spreading to less populous areas (S4 Fig). We did not observe a significant relationship between elevation and epidemic onset (*ρ* = 0.212, *P* = 0.309) (S4 Fig), though elevation did contribute to case incidence, likely modulated by temperature (Fig 3 and S5 Fig). We also found that cholera persistence, estimated as the proportion of weeks with cholera reports, was positively correlated with population size (Spearman *ρ* = 0.61, *P* = 0.001).

**Fig 4:**
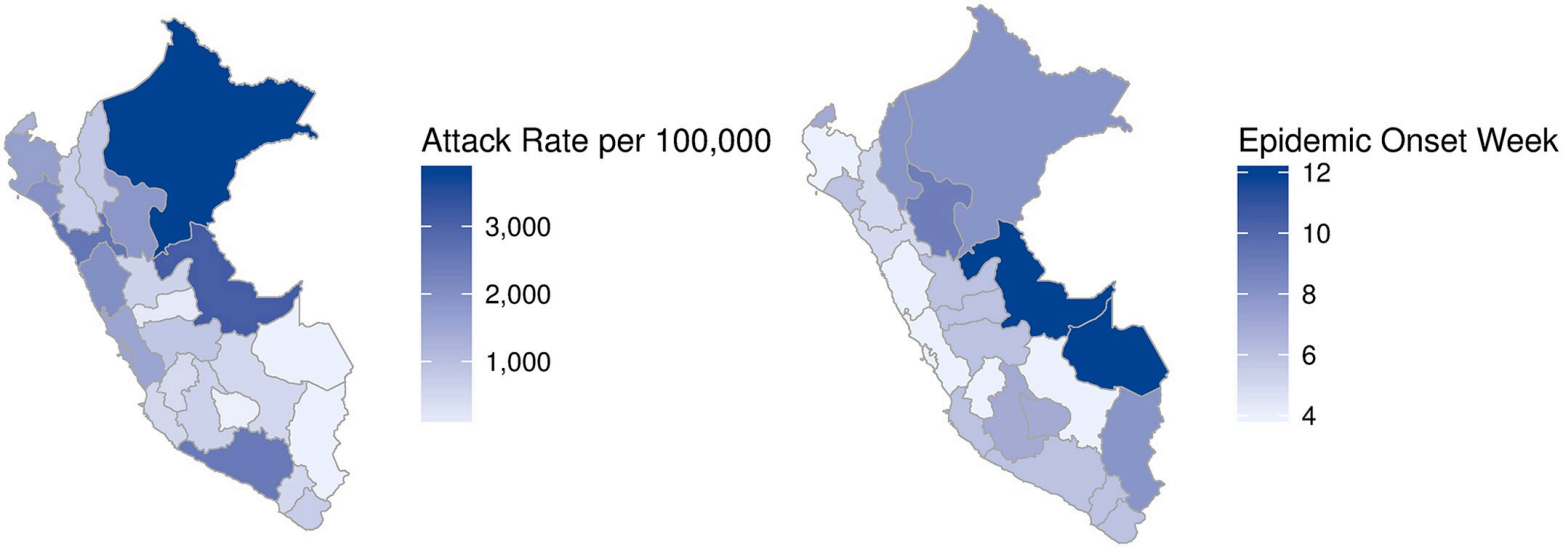
a) Cholera attack rates per 100,000 for Peruvian departments in 1991. The highest attack rates occurred in the jungle region. Coastal departments also showed consistently high attack rates. b) Map showing week of epidemic onset by department. Darker regions experienced a later onset, defined as the first week in 1991 with reported cholera cases. The map was created using the function *admin1_choropleth* using R.

Throughout 1991, coastal departments saw far more cases than the remaining regions (Fig 1). Variability in observed case incidence was related to population size (*ρ* = 0.67, *P* < 0.001), as illustrated by differences in regional attack rates (Fig 4a). However, some of the highest observed attack rates occurred in the jungle and not the more populous coastal cities. For example, Loreto and Ucayali suffered attack rates as high as 3% in 1991. Interestingly, these departments were also among the last to be hit by the epidemic (Fig 4b). Moreover, there was also extreme variation in attack rates within regions. For example, Moquegua saw an attack rate of less than 0.5% in 1991, while La Libertad saw over 2.6%. Both are coastal departments. Further spatial analysis revealed that Moran’s *I* was weak over the course of the epidemic (*P* > 0.08), which indicates that cholera incidence in one department was not strongly correlated with incidence in neighboring departments, likely due to the rapid spread of cholera across the entire territory in a matter of a few weeks. Additionally, as the epidemic progressed we saw greater spatial heterogeneity, with *G* = 0.16 for all seven years of the epidemic (1991-1997).

There was also a clear seasonal trend throughout the course of the epidemic. Cases surged at the beginning of each epidemic year, a pattern that would persist until 1995. We hypothesized that this seasonal trend was a result of environmental drivers, and we chose to assess how fluctuations in temperature might contribute to cholera incidence in each department. Average minimum temperature was correlated with average case incidence over the first three years of the epidemic and correlation was strongest in the coastal departments (Fig 3). The highlands region showed weaker positive correlations, whereas the jungle departments did not show a consistent relationship between temperature and cholera incidence.

**Fig 5:**
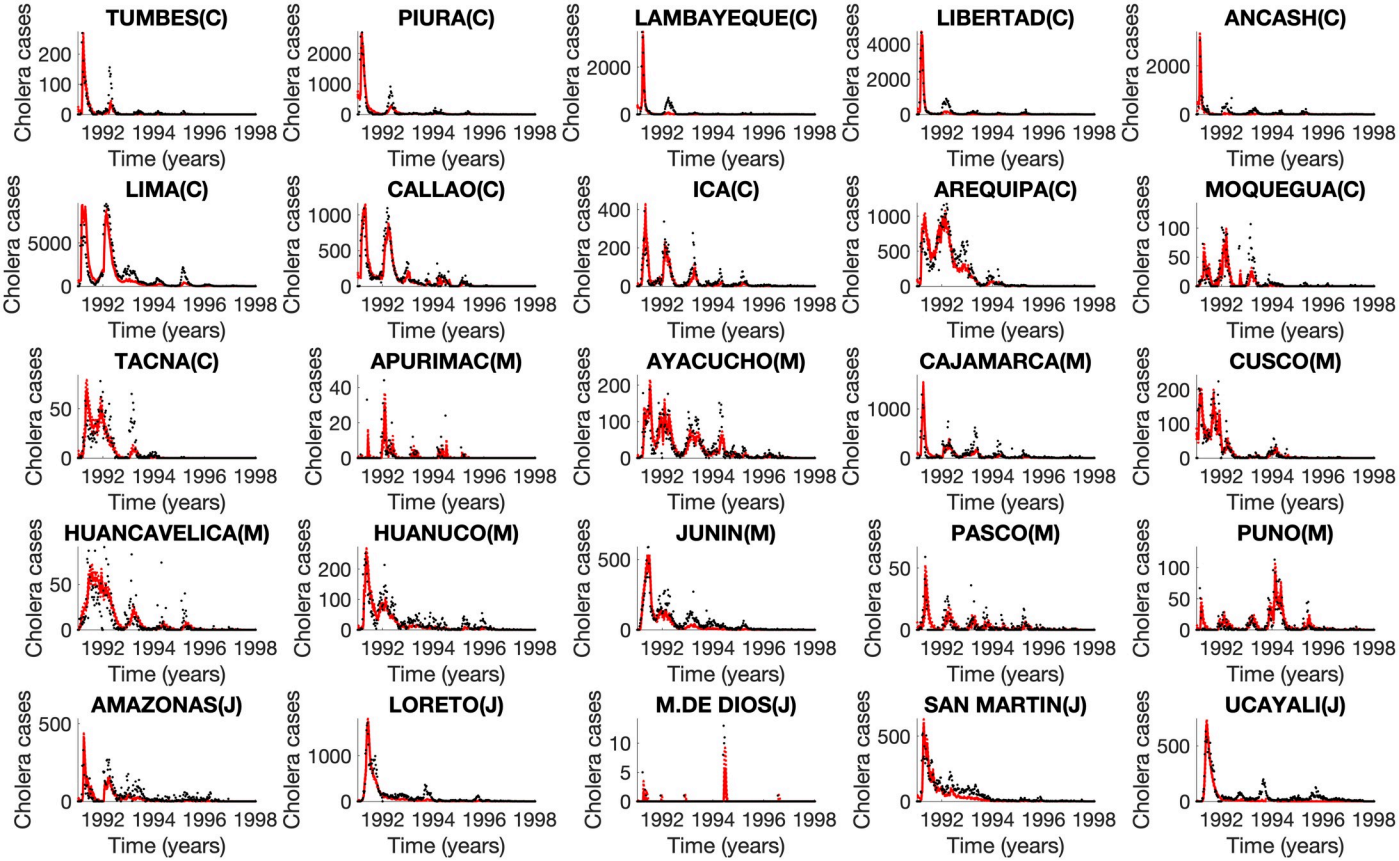
Mechanistic model fits to weekly cholera incidence across 25 departments in Peru, 1991-1997. The black dots correspond to the data, whereas the model mean fit is the red solid line and the dashed red lines correspond to the 95% prediction intervals.

**Fig 6:**
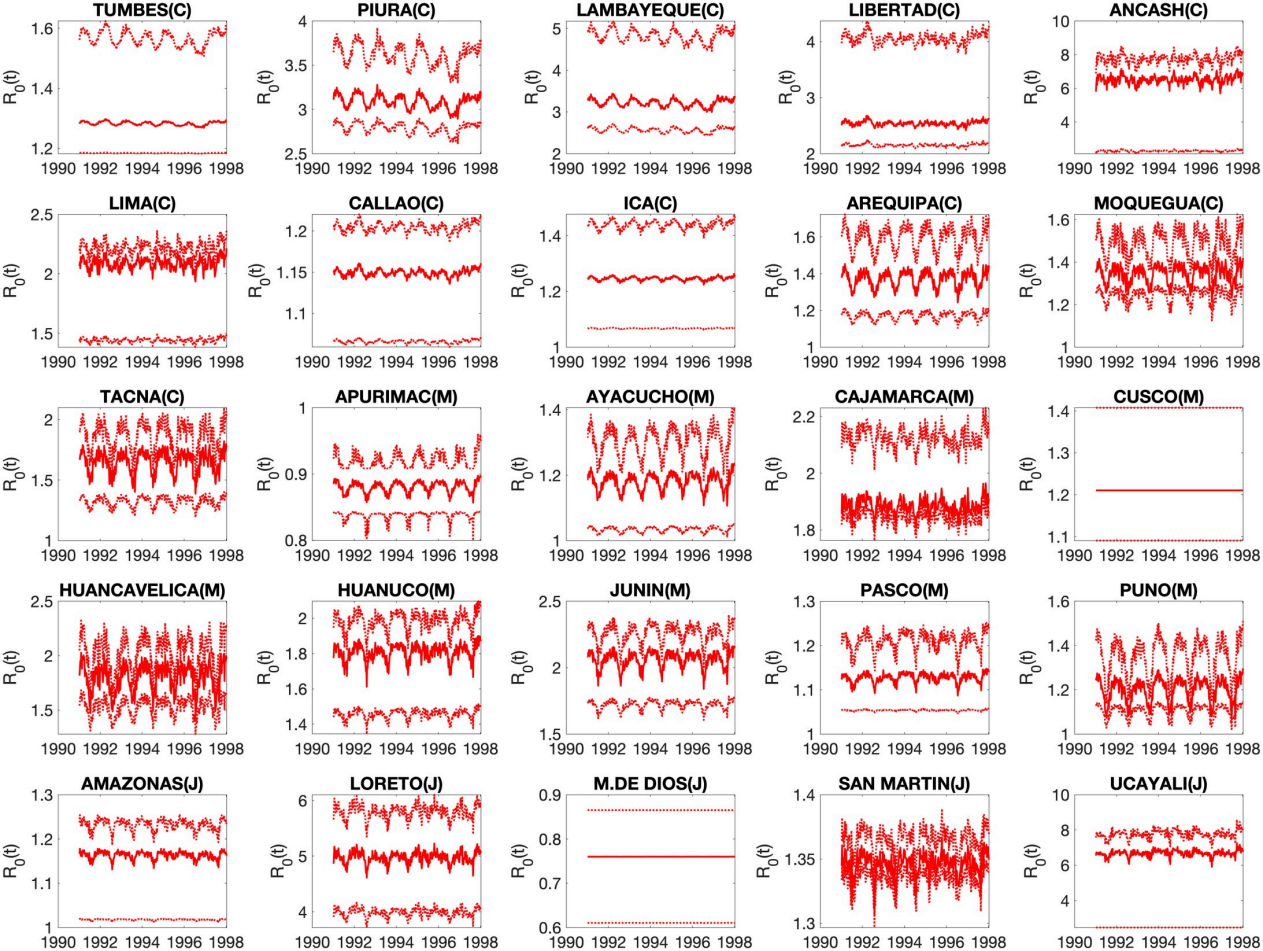
Time-dependent *R*0*s* across 25 department throughout the epidemic (1991-1997). The mean is the red solid line and the dashed red lines correspond to the 95% confidence intervals.

**Fig 7:**
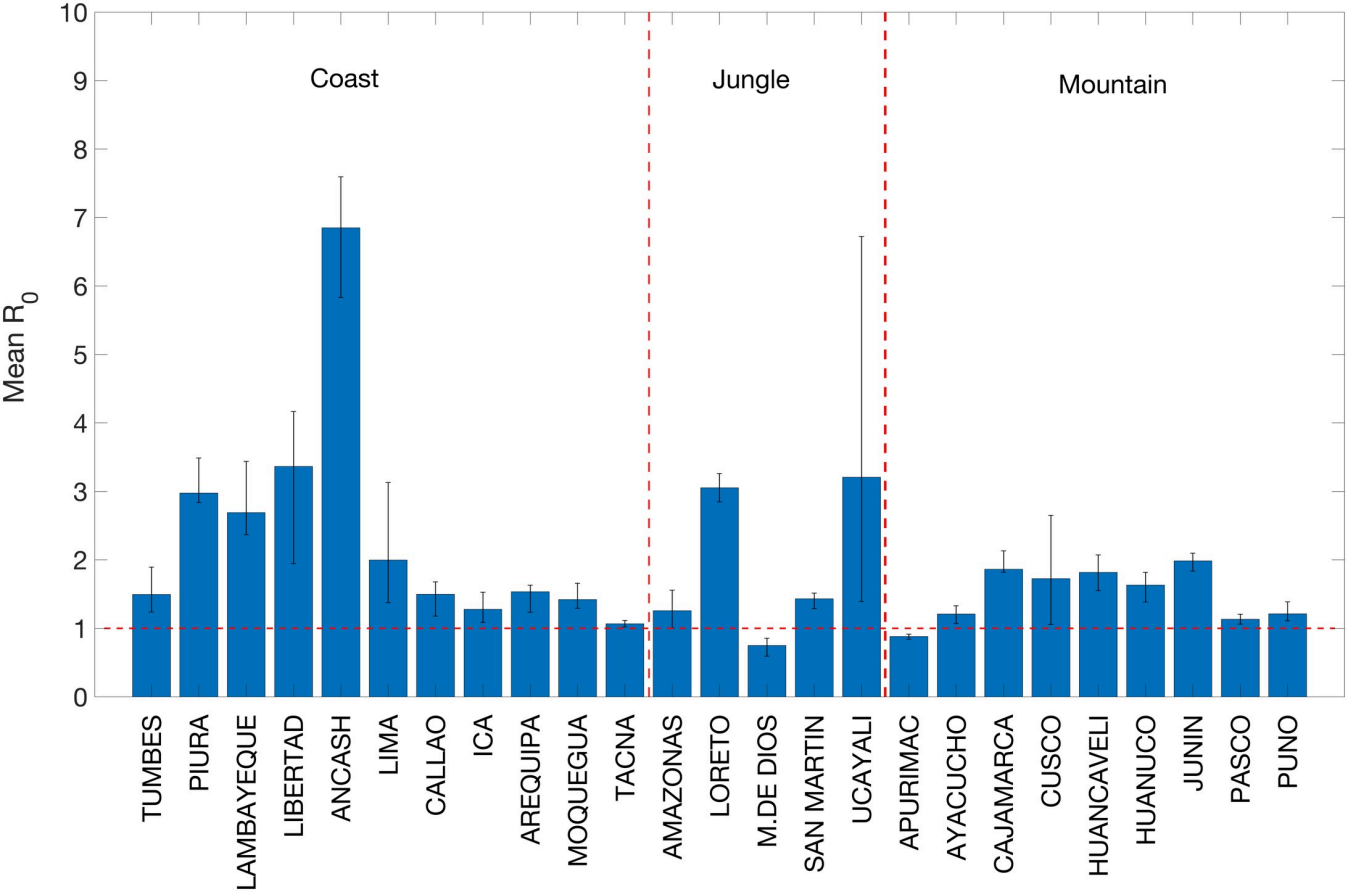
Mean estimates of $R_{0}$ and their 95% confidence intervals across 25 departments in Peru. The horizontal dashed line at 1.0 is shown for reference. The dashed vertical lines separate the departments in the coast, jungle and highlands areas in Peru.

Our mechanistic transmission model yielded good fits to weekly incidence curves across all 25 departments in Peru (Fig 5), allowing us to derive time-dependent $R_{0}\left(t\right)$, mean $R_{0}$ estimates, and estimates of the effective reproduction number across departments. The model showed characteristic seasonal fluctuation in transmission potential (Fig 6 and S6 Fig) as well as in the estimated concentration of vibrios in the environment (S7 Fig). The correlation between model predictions and observations (S8 Fig) was high and on average above 80% across the great majority of the departments (range: 58% to 97%). Across the 25 departments, we estimated $R_{0}=2.1$ (95%CI:, 0.8, 7.3), large enough for sustained transmission. Our department-level mean $R_{0}\text{s}$ showed substantial variability across departments, ranging from 0.8 to 6.9 (Fig 7). Mean $R_{0}$ estimates ranged from 1.1 to 6.9 in the coast, 0.75-3.2 in the jungle, and 0.9-2.0 in the highlands. In particular, the highest mean $R_{0}$ was observed in the coastal department of Ancash (Fig 7). Overall, mean $R_{0}$ was higher for departments that were closer to the coast (Spearman *ρ* = −0.56, *P* = 0.004). We also found that the department-level $R_{0}\text{s}$ were correlated with the overall attack rates (Spearman *ρ* = 0.58, *P* = 0.002), elevation (*ρ* = −0.41, *P* = 0.04), and population size (*ρ* = 0.62, *P* < 0.001). Thus, these results indicate that the epidemic in the coastal region not only exhibited an early epidemic onset based on the timing of the first reported cases, which is consistent with being in close proximity to an aquatic reservoir, but also higher transmission potential relative to the jungle and highlands regions.

**Fig 8:**
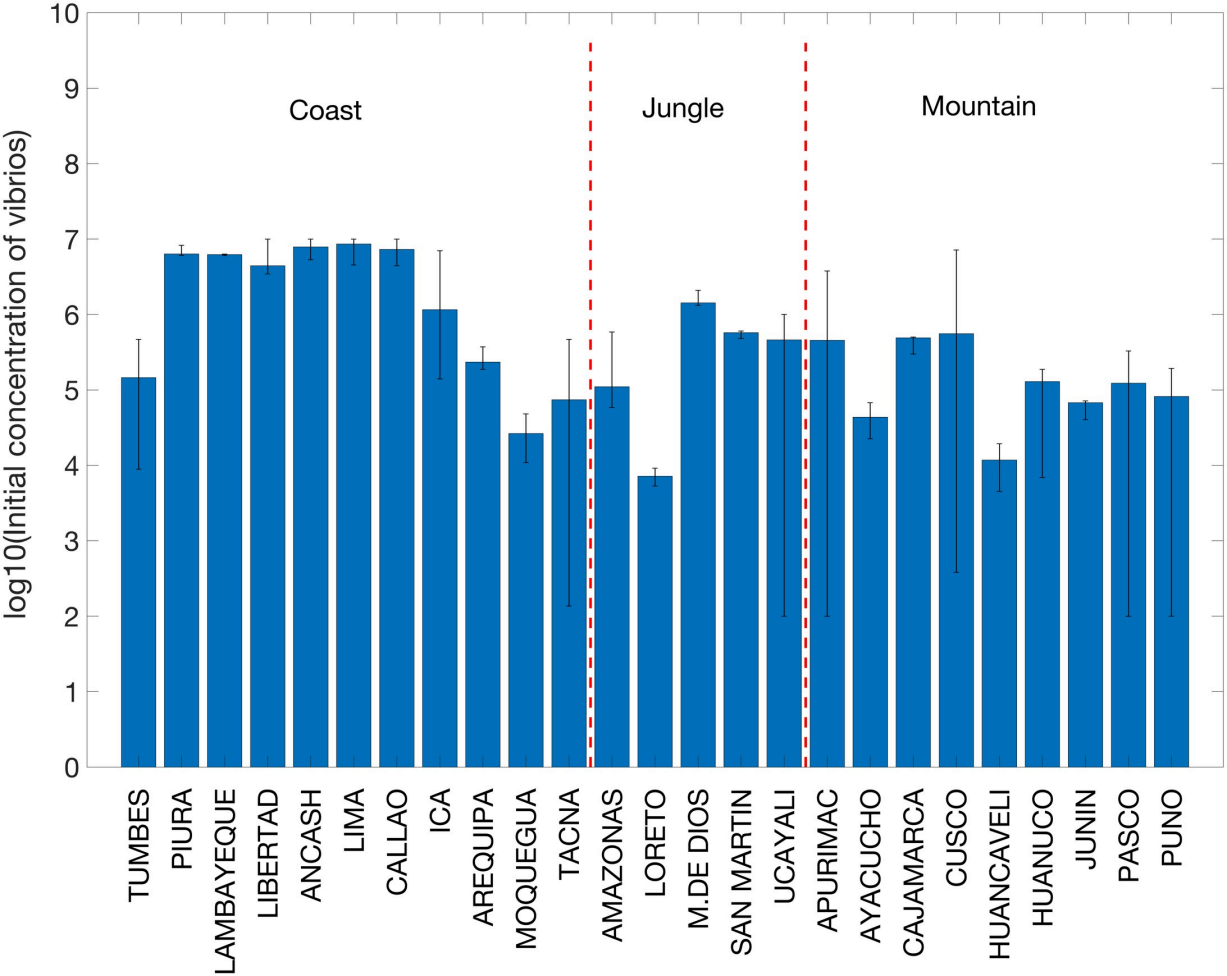
Mean estimates of the initial concentration of vibrios and their 95% confidence intervals across 25 departments in Peru. The dashed vertical lines separate the departments in the coast, jungle and highlands areas in Peru.

We also assessed the variability in estimates of initial concentration of vibrios (*B*_1_) and reporting rates (*ψ*) between departments. We found that the initial concentration of vibrios was higher in coastal departments compared to other departments (log_10_(*B*_1_) = 6.1 vs log_10_(*B*_1_) = 5.2, Wilcoxon test, *P* = 0.02; Fig 8). On the other hand, reporting rates were low, which is consistent with the significant fraction of asymptomatic or mild cases that is associated with cholera infections with the El Tor cholera biotype. Moreover, mean estimates of reporting rates across departments were negatively correlated with illiteracy rates in 1994 (*ρ* = −0.56, *P* = 0.003), possibly indicating weaker surveillance in poorer areas (S9 Fig).

## Discussion

In this paper we have characterized the spatial-temporal dynamics of the great cholera epidemic in Peru (1991-1997) by fitting statistical and mechanistic models to spatially disaggregated weekly incidence time series. Our results shed light on geographic variability in estimates of basic reproduction number ($R_{0}$), initial concentration of vibrios in the environment, and reporting rates. Overall our findings indicate that the initial spread of the epidemic observed in coastal areas aligns with higher $R_{0}$ estimates and concentrations of vibrios in the environment in this geographic region. These findings are consistent with early reports of cholera cases in coastal cities including Lima, Chancay, Chimbote, Trujillo, Chiclayo and Piura [21]. These results suggest that cholera vibrios, autochthonous to plankton in the natural aquatic environment, may have triggered outbreaks in multiple locations along the Pacific coast of Peru before propagating through the highlands and jungle areas [21]. Nevertheless, we cannot rule out that infected travelers arriving from a cholera outbreak area may have contributed to seeding the epidemic [63, 64, 65].

Our department-level estimates of $R_{0}$ in the immunologically naive Peruvian population are generally consistent with the cholera modeling literature. For example, Phelps et al. recently examined cholera epidemics in immunologically naïve populations of 19th century Denmark, and found basic reproduction numbers ranging from 1.7 to 2.6 [63]. Similarly, mean estimates ranged between 1.6 and 3 in the 2010 Haiti outbreak [66, 67, 32], and were also consistently greater than 2 in Yemen (2016) [68]. Other estimates have been reported as high as 5 [69] in Bangladesh (2005) [69], and reached 19.1 and 7.32 in India (2009) and Guinea-Bissau (2008), respectively [70, 30].

Mean estimates for $R_{0}$ were greater than one in most coastal and jungle departments, while estimates were lower in the highlands region (Fig 7). This indicates increased transmission in coastal and tropical climates although available case data were not sufficient to disentangle the contributions of environmental and human-to-human transmission pathways to $R_{0}$. We note that previous studies suggest a large environmental component of transmission in geographic regions neighboring an aquatic reservoir. For example, Mukandavire et al. found that although transmission was driven by environmental contamination in Haiti, only one department-level estimate favored the environmental component of the reproduction number [29], and it coincided with the location of the contaminated river. Conversely, in Zimbabwe, Mukandavire et al. suggests that human-to-human transmission by far outweighed environmental transmission, likely because the country is far from any natural cholera reservoir [28]. Our results suggest higher transmission suitability in coastal areas, which also observed earlier case reports.

The observed spatial-temporal variation in cholera dynamics in Peru is consistent with previous evidence from Mexico, Africa, and Cameroon as well. Studies have found complex environmental factors to be implicated in cholera transmission patterns. For instance, Ngwa et al. used a regression model to identify associations between risk of cholera transmission and environmental variables in Cameroon [71]. They found significant associations between cholera, average daily maximum temperature and precipitation levels. Nkoko et al. studied the Great Lakes Region of Africa from 1978 to 2008 and found that abnormally warm El Niño events corresponded to increases in cholera incidence [72]. This is consistent with hypotheses that El Niño may have influenced the 1991 epidemic in Peru [21]. For example, studies have found significant correlations between cholera incidence and elevated sea surface temperature during El Niño events [73]. Additionally, Latin American countries near other large bodies of water were not affected to the same degree as Peru [74]. The El Niño event may also help explain why cholera is able to persist in the environment without causing endemic infections in a country like Peru [73].

Separating the contributions of human-to-human transmission and environmental transmission was not feasible in the absence of disaggregated case time series arising from each transmission route. More than one possible combination of transmission parameters could have given rise to the Peruvian epidemic curves as noted in another cholera modeling study [54]. Also, we only saw a weak relationship between $R_{0}$ and mean temperature across departments. Finally, it is important to point out that $R_{0}$ is not the sole driver of outbreaks in our cholera model. When the concentration of vibrios in the environment is large, our model can yield substantial outbreaks even for $R_{0}$ values that are below 1.0.

The parameter estimation inverse problem, resulting from this model, is cast as a nonlinear LSP constrained by a system of nonlinear differential equations. In general, a standard approach to identifying parameters from this LSP involves the use of some regularized gradient or Gauss-Newton-type optimization scheme combined with a numerical method for solving the ODE system. Even with regularization, this method is highly unstable [52] due to severe noise propagation aggravated by computational errors at every step of the iterative process. As an alternative to this technique, in our study we design a new problem-specific parameter estimation procedure, which, due to its unique use of incidence data, leads to analytic expressions for *S*(*t*) (the number of susceptible individuals), *I*(*t*) (the number of infected individuals), and *B*(*t*) (the concentration of vibrios in the environment). As such, it no longer relies on numerical solvers for nonlinear differential equations. This allows us to substantially reduce accumulation of computational errors and their magnified impact on the recovered parameters, hence making parameter estimation much more stable and accurate.

Our study is not exempt of limitations. There was substantial underreporting of cases due to prevalence of asymptomatic or mild infections linked to the El Tor biotype [5]. We considered a broad case definition of cholera that includes confirmed and suspected cases [5]. Although our mechanistic model accounts for underreporting of cases, misclassification cannot be ruled out during epidemics. Additionally, the environmental component in our transmission model was only modulated by temperature and did not incorporate potential effects of other environmental variables such as rainfall (see e.g., [75]). Further, additional indicators of environmental transmission may help explain the strong environmental component to transmission in the jungle despite the weak association between temperature and case incidence in this geographic region. Finally, our model did not account for diffusion between departments due to lack of reliable data on movement and connectivity patterns in Peru. However, our spatial analysis revealed relatively weak spatial autocorrelation throughout the epidemic, indicating diffusion was limited (see also [76]).

In summary, the 1991-1997 cholera epidemic in Peru was characterized by distinct waves of transmission through the three geographic regions. $R_{0}$ and initial concentrations of vibrios were substantially higher in coastal areas compared to other regions. Spread throughout Peru was unique compared to other Latin American countries. Using a mechanistic modeling approach that integrates fluctuations in the environmental transmission route, we were able to capture the multiple transmission waves of this epidemic. This methodology could be useful to investigate future epidemics of cholera and could be extended to generate near real-time forecasts and projections of vaccination impacts.

### Disclaimer

This work does not necessarily represent the views of the US government, the NIH, or PAHO/WHO.

## Supporting information

S1 Fig Map of Peru with departmental divisions (created using QGIS). The geography of Peru covers a range of features, from a western coastal plain (yellow), the Andes Mountains in the center (brown), and the eastern jungle of the Amazon (green).
(TIF)

S2 Fig Maps of elevation, illiteracy rate, population size, and mean temperature across 25 departments in Peru. These datasets are available online [44].
(TIF)

S3 Fig Normalized curves of cumulative incidence at the department level, January 1991 through December 1997. The national curve (black dashed line) is also shown for reference.
(TIF)

S4 Fig Spearman (nonparametric) correlations between the week of epidemic onset and (a) population size and (b) elevation (Km). Departments with larger populations tended to have an earlier epidemic onset (*P* < 0.01), while there was no significant relationship between elevation and epidemic onset.
(TIF)

S5 Fig Spearman (nonparametric) correlation between department seven-year cumulative incidence rate and elevation. Departments with higher elevation tended to have a lower incidence rate from 1991-1997.
(TIF)

S6 Fig Effective reproduction numbers across 25 department throughout the epidemic (1991-1997). The mean reproduction number is the red solid line and the dashed red lines correspond to the 95% confidence intervals. The ensemble of cyan curves display the uncertainty in the effective reproduction number.
(TIF)

S7 Fig Estimated mean curves of the concentration of vibrios in the environment and their 95% confidence intervals across 25 departments in Peru. (TIF)

S8 Fig Correlation of the mechanistic model predictions and observations across 25 departments in Peru, 1991-1997. (TIF)

S9 Fig Estimates of the reporting rates and their 95% confidence intervals across 25 departments in Peru. The dashed vertical lines separate departments in coast, jungle and other areas in Peru.
(TIF)

S1 Table (XLSX)
