# Supplementary figures and images for: Spatial dynamics and the basic reproduction number of the 1991–1997 Cholera epidemic in Peru
Source: PLoS Negl Trop Dis. 2020 Jul 14;14(7):e0008045. doi: 10.1371/journal.pntd.0008045 (PMC7360044; doi:10.1371/journal.pntd.0008045)

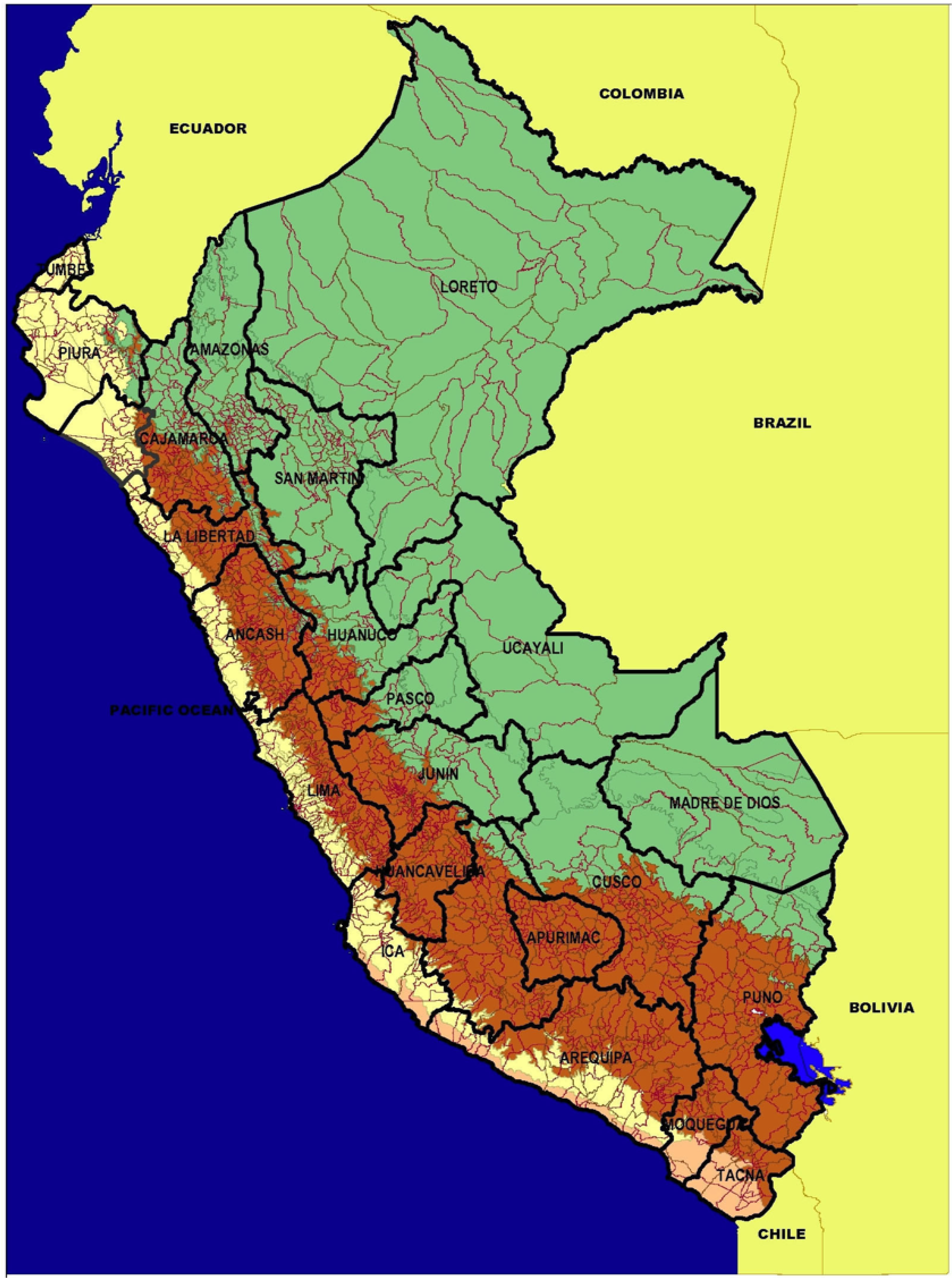

Supplement: S1 Fig — The geography of Peru covers a range of features, from a western coastal plain (yellow), the Andes Mountains in the center (brown), and the eastern jungle of the Amazon (green). (TIF) [file pntd.0008045.s001.tif]

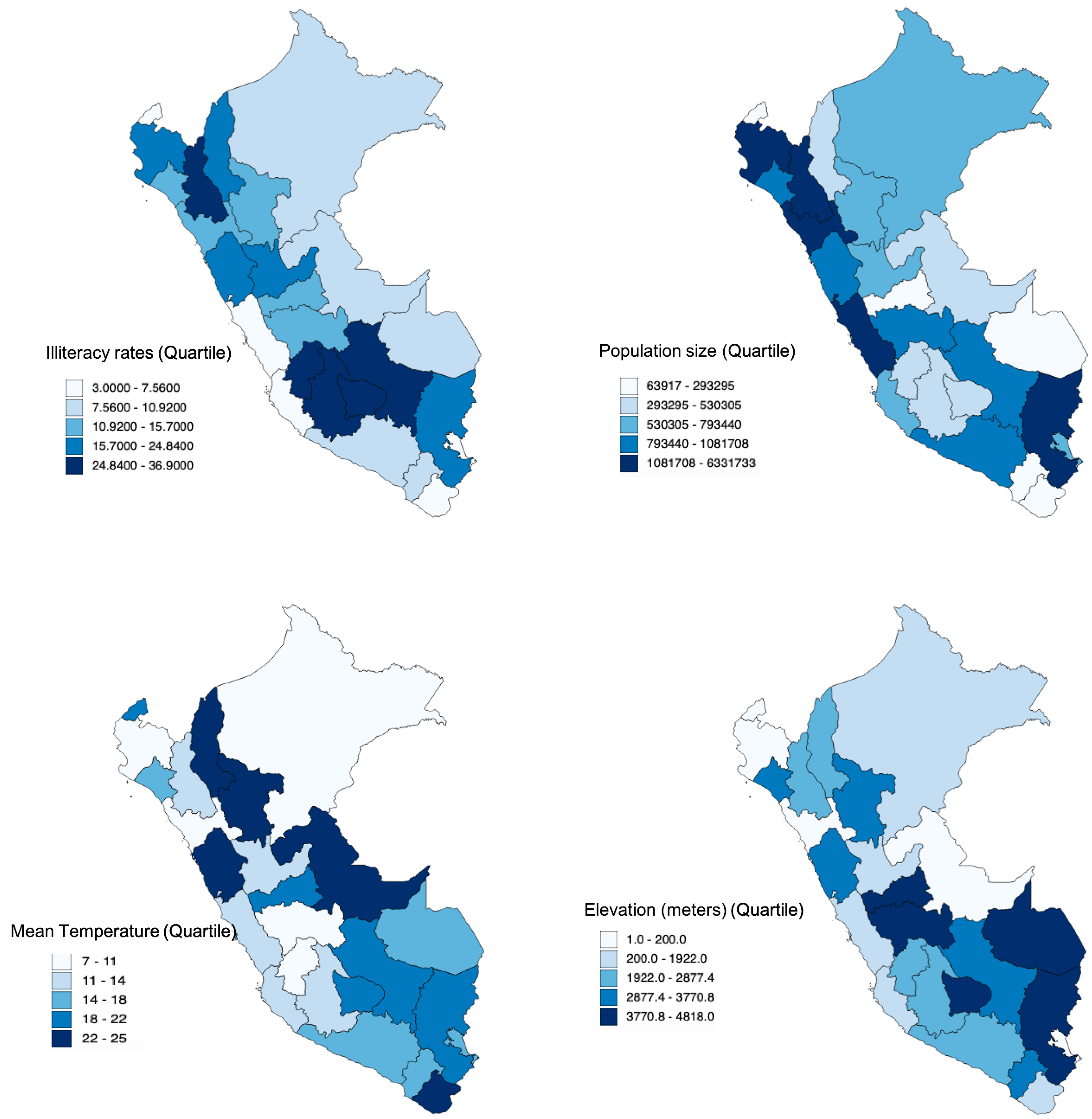

Supplement: S2 Fig — These datasets are available online [44]. (TIF) [file pntd.0008045.s002.tif]

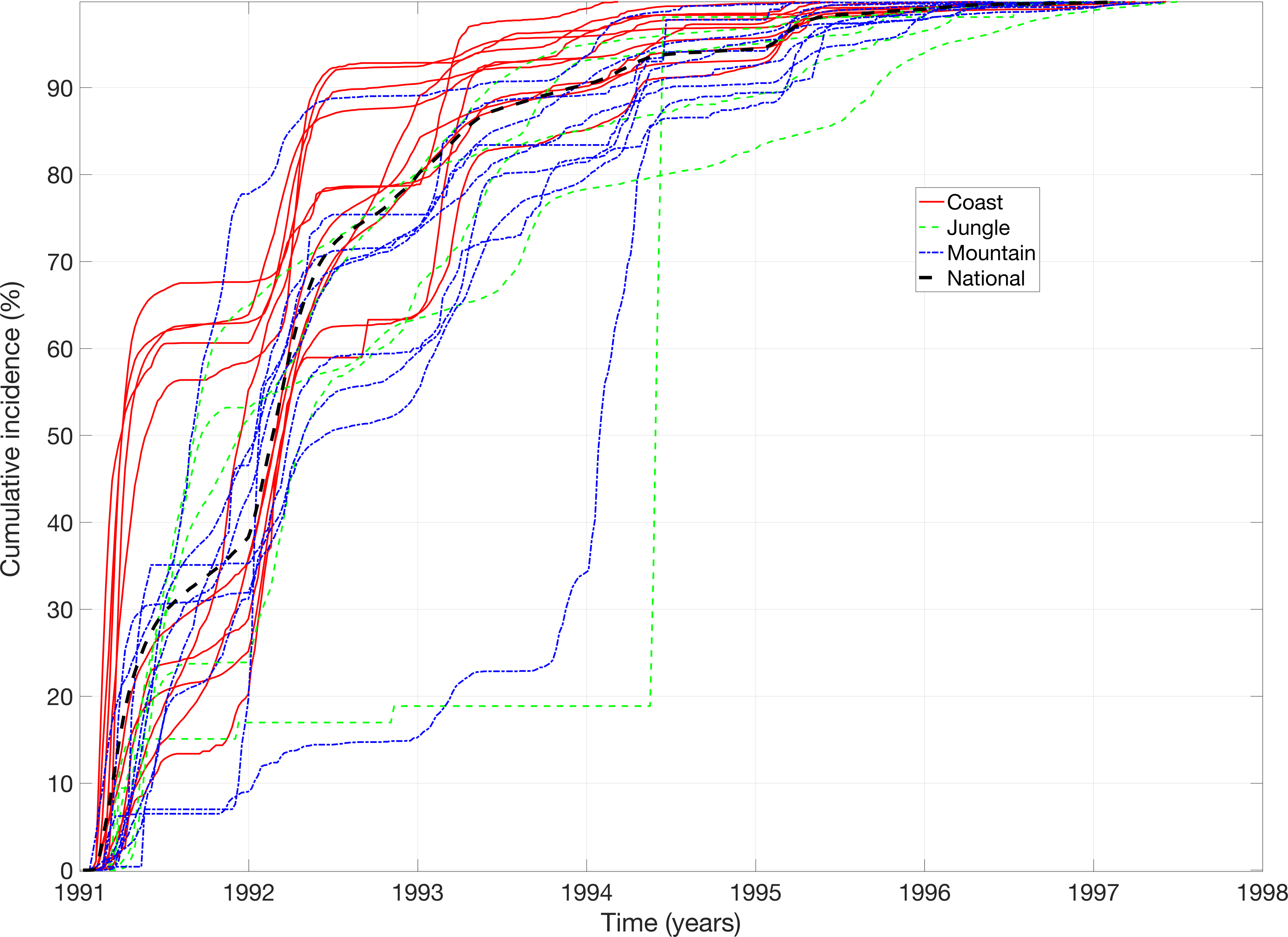

Supplement: S3 Fig — The national curve (black dashed line) is also shown for reference. (TIF) [file pntd.0008045.s003.tif]

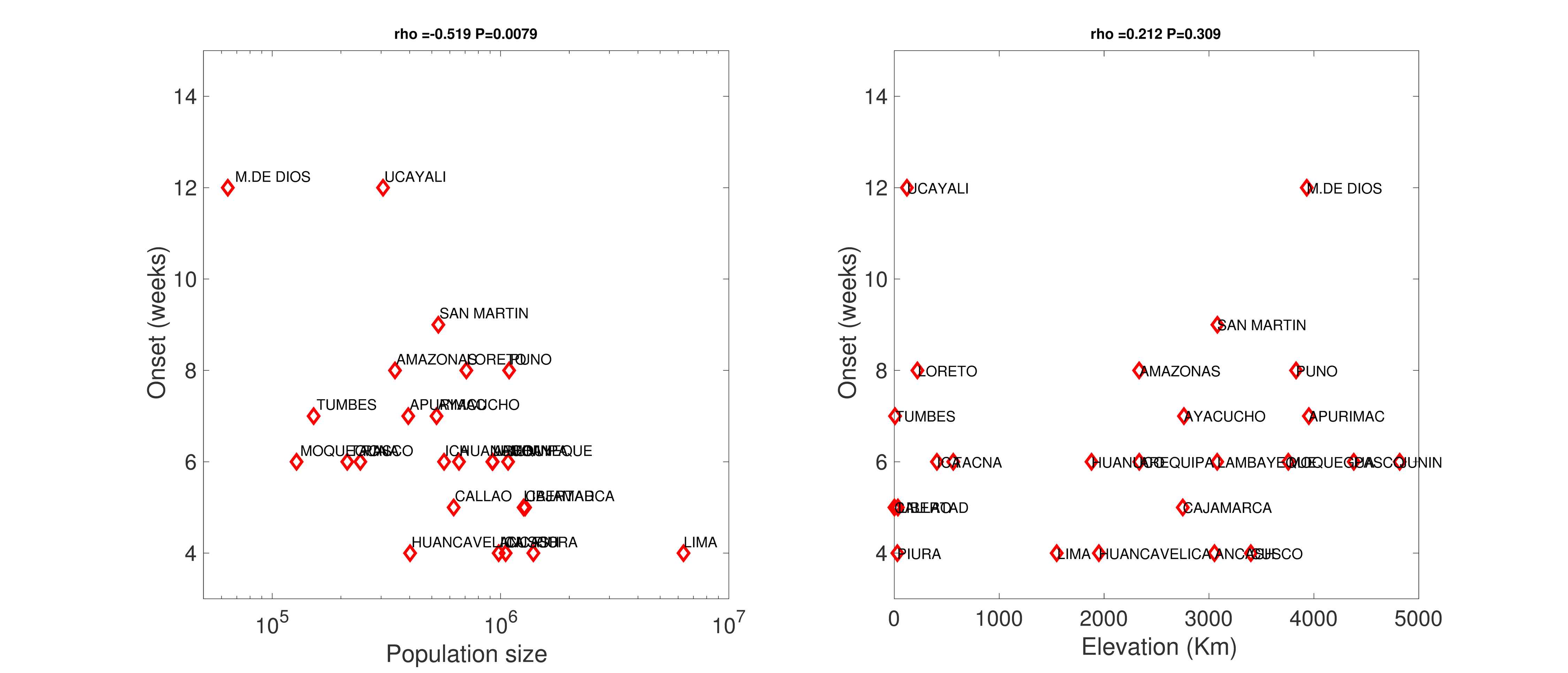

Supplement: S4 Fig — Departments with larger populations tended to have an earlier epidemic onset (P < 0.01), while there was no significant relationship between elevation and epidemic onset. (TIF) [file pntd.0008045.s004.tif]

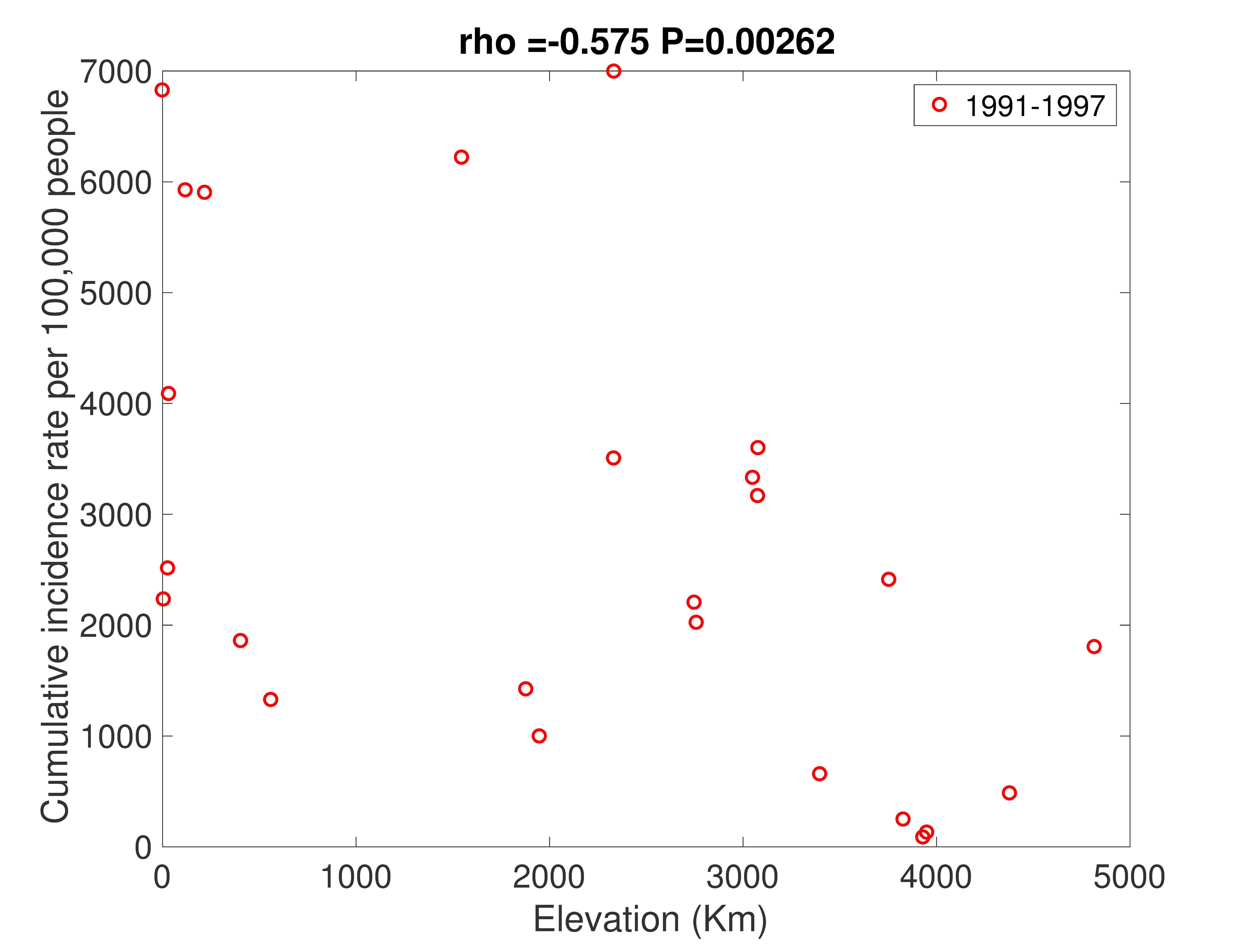

Supplement: S5 Fig — Departments with higher elevation tended to have a lower incidence rate from 1991-1997. (TIF) [file pntd.0008045.s005.tif]

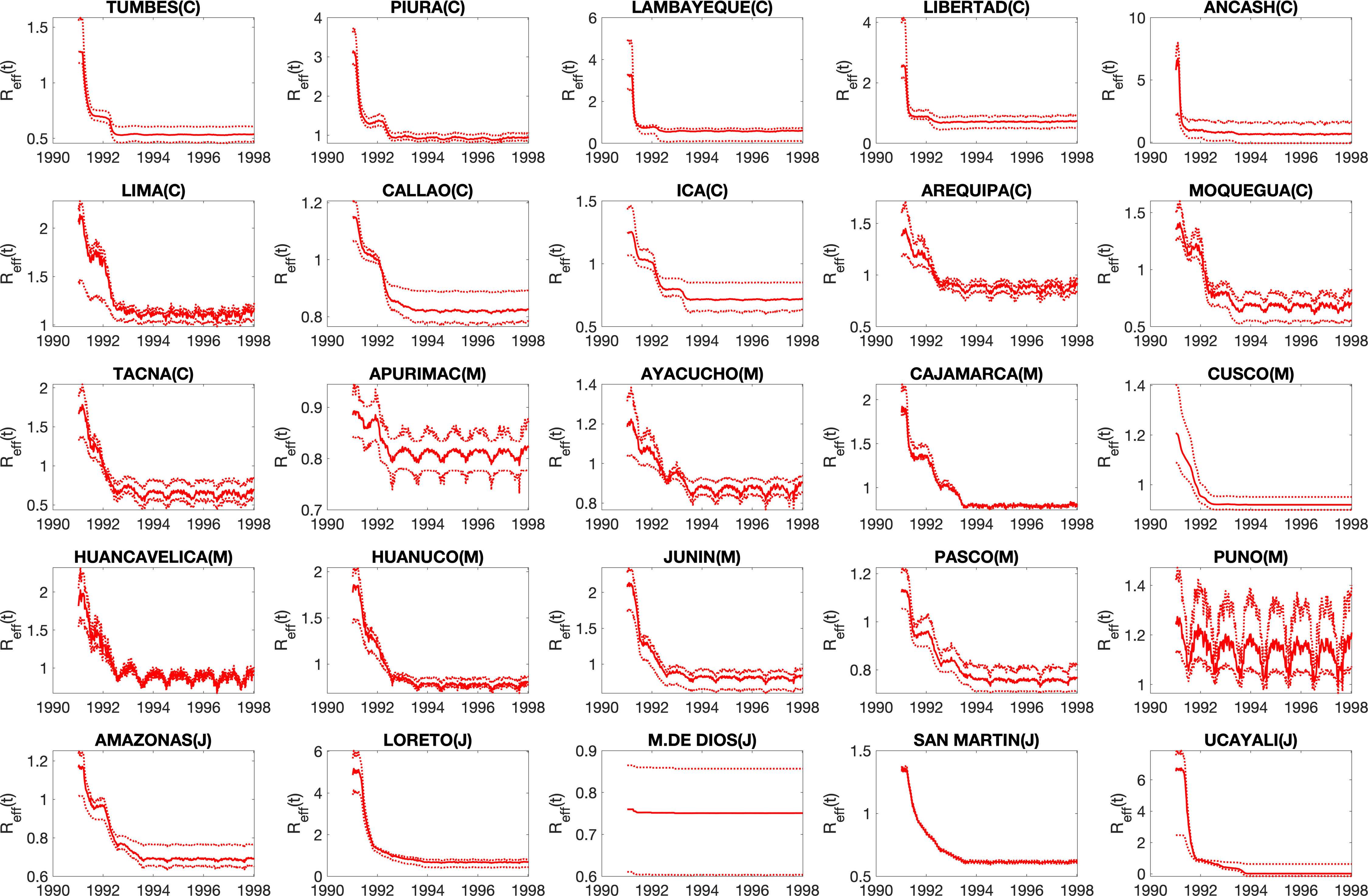

Supplement: S6 Fig — The mean reproduction number is the red solid line and the dashed red lines correspond to the 95% confidence intervals. The ensemble of cyan curves display the uncertainty in the effective reproduction number. (TIF) [file pntd.0008045.s006.tif]

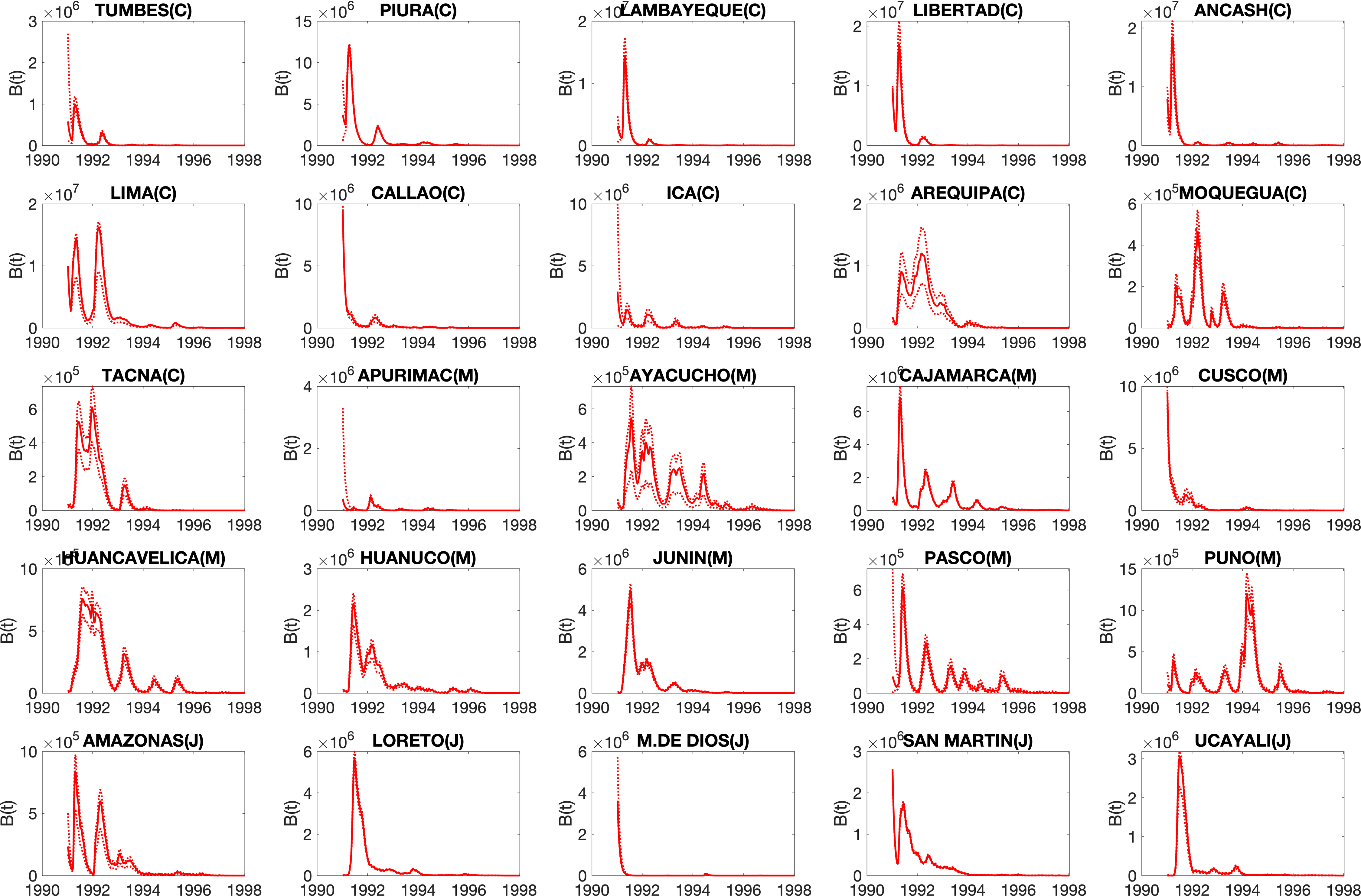

Supplement: S7 Fig — (TIF) [file pntd.0008045.s007.tif]

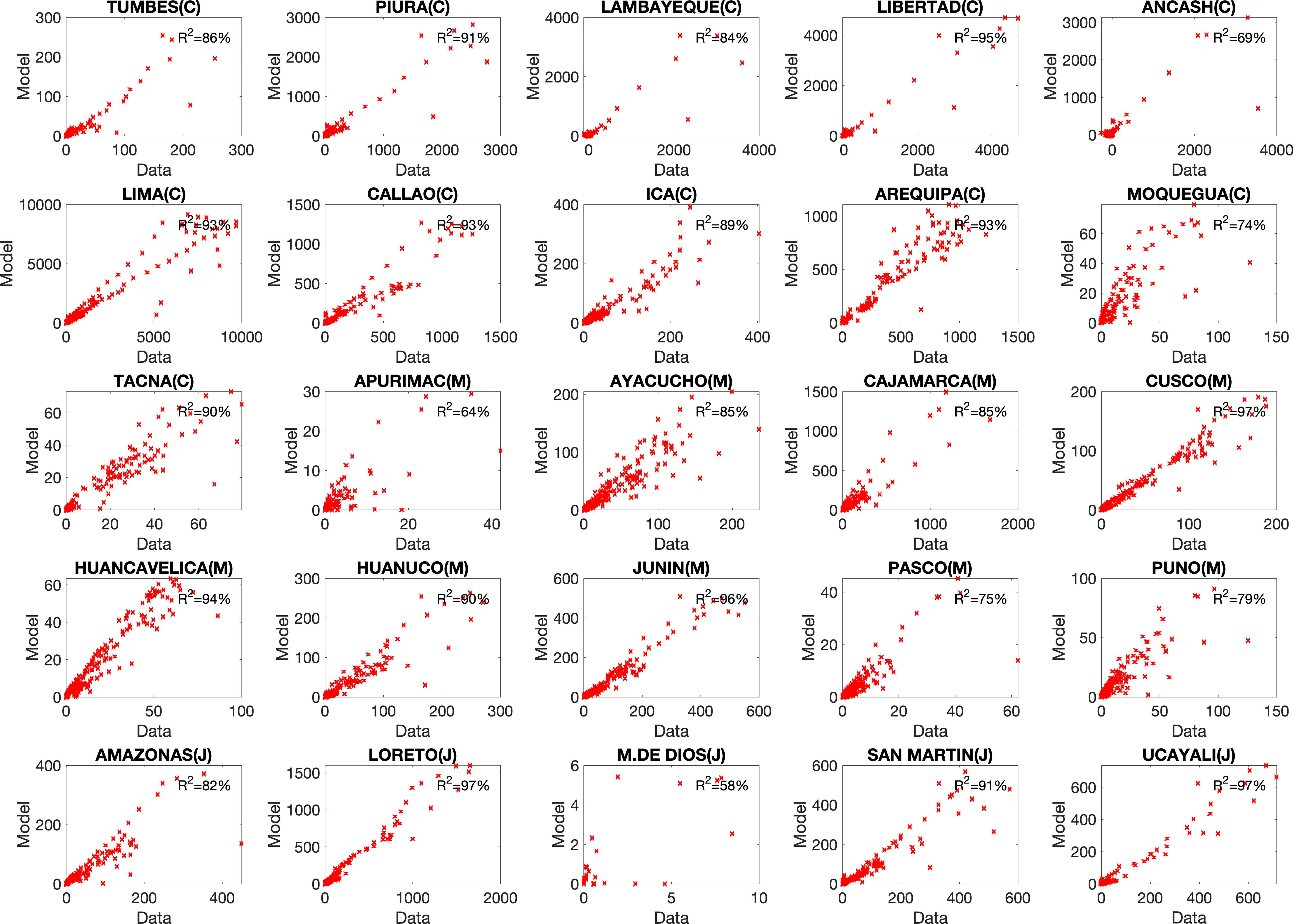

Supplement: S8 Fig — (TIF) [file pntd.0008045.s008.tif]

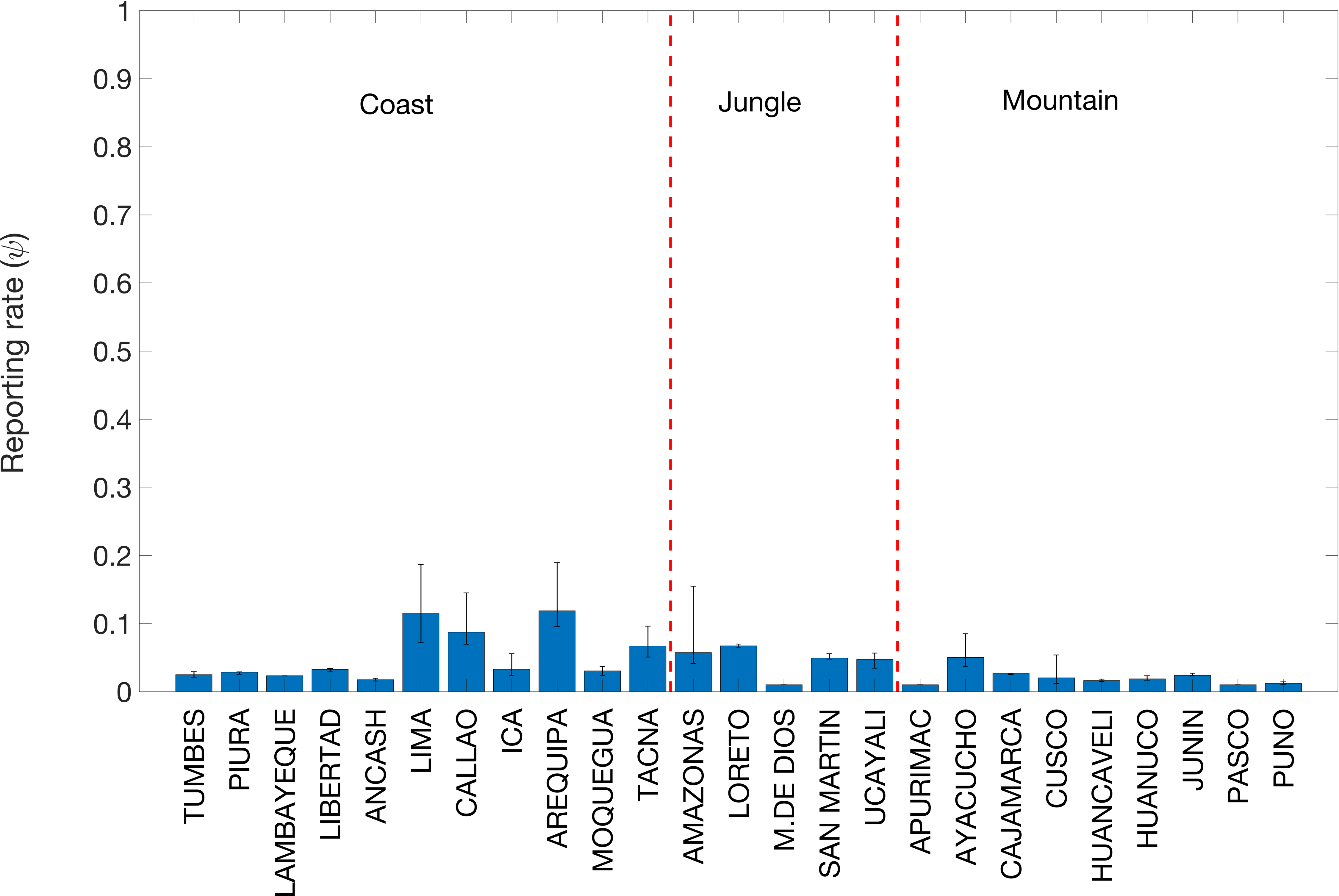

Supplement: S9 Fig — The dashed vertical lines separate departments in coast, jungle and other areas in Peru. (TIF) [file pntd.0008045.s009.tif]
